# Aptamer-Based Analysis: A Promising Alternative for Food Safety Control

**DOI:** 10.3390/s131216292

**Published:** 2013-11-28

**Authors:** Sonia Amaya-González, Noemí de-los-Santos-Álvarez, Arturo J. Miranda-Ordieres, Maria Jesús Lobo-Castañón

**Affiliations:** Departamento de Química-Física y Analítica, Universidad de Oviedo, Julián Clavería, 8, Oviedo 33006, Spain; E-Mails: amayasonina@uniovi.es (S.A.-G.); santosnoemi@uniovi.es (N.S.-Á.); amir@uniovi.es (A.J.M.-O.)

**Keywords:** aptamers, allergens, emerging contaminants, food safety, natural toxins, pathogens

## Abstract

Ensuring food safety is nowadays a top priority of authorities and professional players in the food supply chain. One of the key challenges to determine the safety of food and guarantee a high level of consumer protection is the availability of fast, sensitive and reliable analytical methods to identify specific hazards associated to food before they become a health problem. The limitations of existing methods have encouraged the development of new technologies, among them biosensors. Success in biosensor design depends largely on the development of novel receptors with enhanced affinity to the target, while being stable and economical. Aptamers fulfill these characteristics, and thus have surfaced as promising alternatives to natural receptors. This Review describes analytical strategies developed so far using aptamers for the control of pathogens, allergens, adulterants, toxins and other forbidden contaminants to ensure food safety. The main progresses to date are presented, highlighting potential prospects for the future.

## 1. Introduction

Foodborne diseases represent a global public health challenge. Each year, millions of people get sick, and some even die as a result of the ingestion of unsafe food and water. The so-called globalization of the food production, processing and distribution, further complicates the problem. Because of the increase in consumers' concerns about what is in their food and its relation with health, control over the safety and quality of food has become tighter, particularly in developed countries where food availability problems are much lower. In fact, both USA and EU have surveillance systems with dissimilar level of coverage. The reporting of investigated foodborne outbreaks is mandatory since 2005 in EU. Nevertheless, large foodborne outbreaks have occurred in recent years. The control of these outbreaks is a multidisciplinary task. Undoubtedly, the availability of more powerful and cheaper analytical methods to detect potentially hazardous components in foods would help to reduce the social and health burden of these food safety issues [1].

Analytical Chemistry is thus challenged to respond to the complex dimension of food security by developing new analytical detection approaches for specific hazards associated to food before they become a problem. A key issue to this is the development of new receptors with enhanced affinity to a given target, which can be used in the development of fast, reliable and sensitive chemical sensors and analytical assays.

Aptamers, nucleic acids with appropriate secondary structures that function as ligands, are emerging as new analytical reagents, which can be coupled with different transduction systems. Possibly, the most important advantages of aptamers from an analytical point of view are the high affinity and binding specificity for their targets, ease to be labeled with different reporter molecules, and their relatively low production cost, which make them ideal reagents for the development of sensors. Although aptamer applications are dominated by clinical or medical diagnostics market so far, first steps have already been taken for the application of aptamers to ensure food safety. The purpose of this review is to provide an overview of the variety of aptamer-based analytical strategies developed so far for the detection of a plethora of analytes whose presence in foods poses some threat to human health. We highlight the main progress and challenges related to control of microbial contamination, by far the most serious problem in food poisoning, as well as natural toxins and other contaminants present in food, either accidentally or deliberately (adulterants), including the presence of allergens which may be not acceptable to certain consumers. In each case, we identify current and emerging issues to the specific subject matter and the potential solution that aptassays offer, without attempting to be exhaustive but trying to choose relevant examples for every distinct strategy or method with applications in food analysis. Readers are referred to recent reviews for a more comprehensive overview of aptamer-based assays [2,3].

## 2. Pathogens

Foodborne zoonotic diseases are infections and diseases naturally transmissible between animals and humans through contaminated foodstuff. They are the main source of foodborne outbreaks. Infectious agents include microorganisms such as bacteria, viruses or parasites and abnormal proteins, prions. In spite of the progressive implementation of good agricultural and handling practices as well as educational programs, the incidence of zoonotic diseases is still high. More than 300,000 cases have been reported in the EU in 2011, with about 300 deaths. In the USA, an impressive number of 47.8 million cases with more than 3,000 deaths are recently estimated from confirmed cases over the period 2000–2008 in the population under surveillance (15% of the total). Over the years some pathogens have been eradicated or minimized but others are continuously emerging. Microorganisms unknown or not considered as a cause of zoonotic disease are being linked to some investigated outbreaks. Foodstuffs previously not associated to these illnesses are now sources of microbial hazards [4].

The problem of foodborne pathogens is even more remarkable because of the low threshold of viable organisms required to trigger a life-threatening illness. So, the selection of the appropriate method of detection is of paramount importance. Reference methods involve time consuming cell culture, and food contamination alerts have to be solved in shorter times. Consequently, a continuous progress in the field of the so-called “rapid methods” is being done by incorporating novel technologies from different disciplines such as DNA-based receptors and nanoparticles [5].

**Figure 1.**
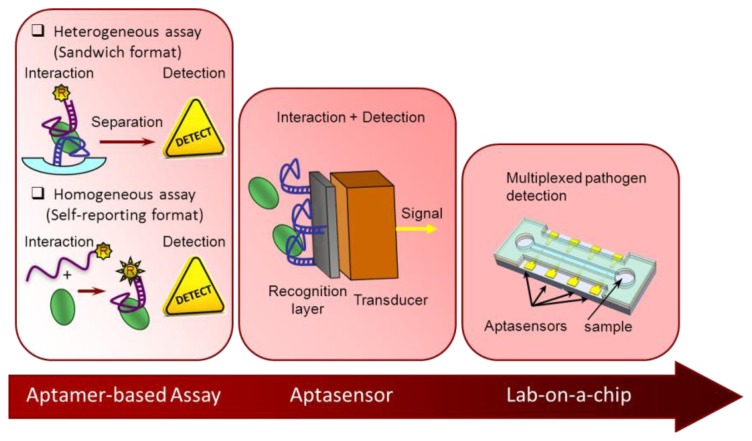
Schematic overview of the different approaches for aptasensing according to their level of integration.

There are several strategies to obtain aptamers that recognize pathogens, either targeting the selection process to a specific part of the pathogenic cell or the whole cell [6]. In spite of the complexity of the process, a noticeable increase in the number of aptamers against pathogens is observed in the last couple of years. An on-line search (from Scopus and Web of Knowledge databases) using as keywords SELEX and pathogen resulted in 74 and 40 hits, respectively for the years 2004–2013, with about half of them published after 2012 (49% or 57%, respectively). No older articles were recovered, though some have been found in detailed revisions [7]. Importantly, not all of those pathogens for which aptamers have been developed have their own aptamer-based detection methodology. In this section the most relevant advances and trends are compiled, classified according their degree of automation and system integration (Figure 1).

In the same way that antibodies are used as analytical reagents in immunoassays, aptamers can be used in the development of analytical assays based on the observation that in a system containing a specific aptamer and the analyte (target), its distribution between bound and free forms is quantitatively related to the target concentration. Adapting ELISA assays to aptamer receptors is the easiest way to develop an aptamer food assay in order to be marketed. This format assay is useful for the determination of macromolecular targets that can be simultaneously bound to two aptamers without steric hindrance. An aptamer (the capture aptamer) is immobilized on a solid surface, *i.e.*, a plate, magnetic particle, *etc.*, which specifically binds the analyte in the sample. An excess of a second labeled aptamer (detection aptamer) is then added, and after incubation and washing, the signal from the bound detection aptamer is measured and related to the analyte concentration in the sample. Aptamers are usually tagged with a small molecule typically biotin, fluorescein or digoxigenin to introduce an enzymatic label through enzyme conjugates (streptavidin- enzyme [8] or Fab antibody fragments- enzyme). This implies an additional step that is not required when enzyme-labeled antibodies are used. To the best of our knowledge conjugates with antibody fragments have not been applied to the field of pathogen detection yet. Since direct labeling of aptamers with enzymes is not readily available, this drawback has to be overcome using other labels. Fluorophores such as FAM (carboxyfluorescein) [9] or quantum dots (QD) [10] can be attached to DNA sequences that, therefore, directly act as a detection aptamer in sandwich assays. These aptamer-based assays usually employ one or two specific aptamers. Nevertheless a collection of aptamers directly arisen from SELEX is proposed for detecting *Francisella tularensis*. This pool of “polyclonal aptamers” is used for both capture and detection, in such a way that a specific aptamer can act as primary or secondary aptamer. Consequently, a variety of different ternary complexes are formed [8]. This strategy rules out the need for time-consuming thorough studies to find the best binder but the overall sensitivity may be somehow lower than that of a pure solution of the leading aptamer. Among all the above-mentioned sandwich assays for pathogens [8,9], only one of them was applied to food samples. It is based on a QD-tagged aptamer for the detection of *Campylobacter jejuni* in artificially contaminated diluted chicken juice, ground beef extract and 2% fat milk, with limits of detection ranging from 10–250 cfu/mL without pre-enrichment [10].

A forward step is the development of self-reporting aptamer-based assays. This strategy relies on the modulation of a measurable property of the label directly attached to the aptamer by the ligand-induced fit that aptamer experiences. In this way, direct monitoring of bound fraction, without prior separation of bound and free tracer, is achieved. Electrochemically active or fluorescent molecules are commonly used for this purpose [11]. Besides these classical tags and taking advantage of the nucleic acid character of aptamers, DNAzymes can be readily incorporated during the chemical automated synthesis of the aptamer. These non-protein enzymes have a mild enzymatic activity that was useful for the chemiluminescent detection of *Salmonella paratyphy A*. In this case, a DNAzyme with peroxidase activity after binding the hemin cofactor is used, and the recognition event does not trigger the enzymatic activity by itself. The DNAzyme-aptamer conjugate wrapping single-wall carbon nanotubes (SWCNTs) is released when both hemin cofactor and target are added. Spiked tap water samples were analyzed, achieving a limit of detection of 10^4^ cfu/mL [12].

The need for labeling is overcome by using an inherent characteristic of nucleic acids: their amplifiability. A RNA aptamer is used as detection aptamer upon capturing the target *E. coli* with an antibody (mixed sandwich) on magnetic beads. Relatively mild release of the aptamer at 80 °C for 15 min avoids cell lysis and the concomitantly release of nucleic acids from the target pathogen. Subsequent detection of the RNA-aptamer by real-time reverse-transcription PCR showed a broad concentration range with a limit of detection of 10 cells/mL of culture sample in the absence of other pathogens. Mixtures of pathogens diminish the detectability about one order of magnitude [13].

The availability of biosensor devices for portable, point-of-need and rapid analysis is perhaps the most desirable approach and so, it is an increasing tendency in analytical chemistry. Aptamers are excellent choice for use as recognition elements in the selective layer in direct contact with the transducer. The most straightforward design is that in which the interaction between the aptamer and the analyte causes a change in the local environment of the transducer surface that can be directly detected by it, so that the recognition event can be converted into a measurable electronic signal. Electrochemical transducers are among the most often used in this design. Two label-free approaches stand out. On one hand the simplicity and the short time response of potentiometric sensors was successfully applied to pathogen (*E. coli*) detection in milk and apple juices with minimal sample pretreatment (filtration). Although the working principle is not clearly understood, the sensor response, that is the potential through a SWCNT-aptamer layer, is stable and reproducible, the sensor is reusable for a limited number of cycles and the limits of detection achieved are excellent even without pre-enrichment steps [14,15]. These features make it very attracting to reach the market. On the other hand, a common problem among non-culture methods is the differentiation of viable and non-viable bacteria. This false-positive issue was addressed recently for *Salmonella* using impedimetric transduction on gold screen-printed electrodes modified with a self-assembled monolayer of a panel of aptamers. Although viable bacteria are distinguishable from non-viable ones, the method has to probe its usefulness in real samples [16]. Alternatively, aptamers can be functionalized with different molecules, acting as signal reporters. Both optical [17] and electrochemical transducers [17,18] have been combined with labeled aptamers for pathogen detection. Two different strategies hold a large potential for food safety control: sandwich indication and determination by displacement. In the sandwich format, the binding of the analyte to the sensing surface including a specific receptor is followed by the interaction with a second labeled aptamer. Thus, a fiber-optic biosensor was proposed as a novel platform for *Listeria monocytogenes* detection. In this study, anti-*Listeria* antibodies were used as capture molecules and aptamers, against an invasive protein of the pathogen, conjugated to a fluorophore as reporters. This sensor has been applied to the detection of *Listeria monocytogenes* in spiked ready-to-eat samples after a long pre-enrichment step [17]. By contrast, displacement assays rely on immobilization of the aptamer in its duplex form with the complementary strand. Depending on which strand is immobilized and/or labeled several assay types are possible resulting in signal-on or signal-off devices [19]. In the case of pathogens, the complementary strand is anchored to the surface while aptamer is partially hybridized to it. This way, the presence of the pathogen causes the release of the aptamer that is thermodynamically driven by the superior stability of the aptamer-target complex over the partial duplex. When none of the strands carry a label, a further re-filling step with a fully complementary labeled-strand is needed. To facilitate the electrochemical detection, the refilling strand can be labeled with a redox active molecule at the end adjacent to the electrode surface. However, in a pathogen biosensor for *E. coli* detection a biotinylated strand was used. In that case, an enzyme conjugate has to be added to generate the analytical signal, so the label necessarily was positioned at the far end with respect to the electrode surface [18].

Both types of sensors offer great potential to quantify pathogens in foods, although the displacement strategy is a more time-consuming alternative, especially when a re-filling step that needs further enzyme incorporation is added.

Multiplexing capability is another desirable feature, since screening each sample for a single pathogen is very laborious, time-consuming and costly. High-throughput technologies such as DNA microarrays have a tremendous potential for biological defense and food/environmental analysis. However, current aptamer microarray development is clearly biased to biomedical applications such as molecular diagnosis or drug discovery. A few examples of limited multiplexing are directed to pathogen analysis in food. For the simultaneous detection of pathogens, sandwich assays using aptamer-modified magnetic particles facilitate the separation steps involving preconcentration. This platform has been combined with the use of reporting-aptamers conjugated to fluorescent labels such as lanthanide-doped upconversion nanoparticles, which exhibit low light-scattering background and induced autofluorescence, resulting in very adequate assays for food matrices. In this way, the simultaneous detection of *Salmonella typhimurium* and *Staphylococcus aureus* in water samples with limits of detection of 5 and 8 cfu/mL was successfully carried out [20]. Even though some level of automation is possible, this kind of bioassays remains laborious, time-consuming and expensive, especially when different pathogens have to be measured. Therefore, there is a need for integrated platforms that could enable rapid and simultaneous detection of different pathogens in numerous samples. As a response, lateral flow and lab-on-a-chip devices are emerging between the aptamer-based methods for food analysis. A method combining both trends, multiplexed detection and lab-on-a-chip, was designed on a PDMS/paper/glass chip that detects *Salmonella* and *S. aureus*. Fluorescently-labeled aptamers are bound to graphene oxide (GO), which quenches its fluorescence. In presence of the pathogenic cell aptamers are released from the GO surface and the fluorescence increases [21].

Prions are a different class of pathogens that are attracting much attention since it is believed that they could trigger a variant of Creutzfeldt-Jakob disease in humans after consuming infected beef products. Several aptamers have been raised against these infectious proteins but all analytical methods were directed to biological samples [22].

## 3. Natural Toxins

Natural toxins are a wide range of molecules generated by fungus, plant or microbiological metabolism that have toxic effects on humans or other vertebrates. The poisonous effects of some of these molecules can be acute even at very low doses. The most important group of natural toxins is mycotoxins, a chemically heterogeneous group of fungal origin. Although the feasibility of high intake of mycotoxins is low in developed countries due to diet variety and strict regulations, some authors consider them as the major chronic dietary risk problem, higher than other contaminants or pesticides. Their formation is unavoidable and the complete removal technically unattainable. It is estimated that about 25% of crops are contaminated by mycotoxins [23].

The chemical diversity as well as the large variety of possible matrices along with their usual trace concentration is a challenge for analytical methods. In addition to this, worldwide safe levels have not been established yet. As an example, the maximum levels of aflatoxin in force in the EU are lower than those recommended by the *Codex Alimentarius*. Currently EU is evaluating the unusual possibility of aligning the values with those of the *Codex*, that is, increasing the safety levels.

In recent years, the detection of not only mycotoxins but also other natural toxins like abrin, ricin, bacterial enterotoxins or botulinum toxin has attracted considerable attention because of their use as chemical warfare agents. Immunoassays are the gold standard for their determination. However, the development of antibodies for these molecules, most of which are low-molecular weight and therefore non-immunogenic molecules, requires conjugation to protein carriers to confer them immunogenicity and the use of animals involves ethical considerations, as these compounds are highly toxic. Aptamers arise as an alternative to the conventional antibody-mediated biotoxin determination.

Although various approaches have been developed for aptamer-based toxin detection [24], there are still many compounds that lack their aptamer or aptamers that lack analytical application. We will focus on the most relevant ones with application in food matrices.

**Figure 2.**
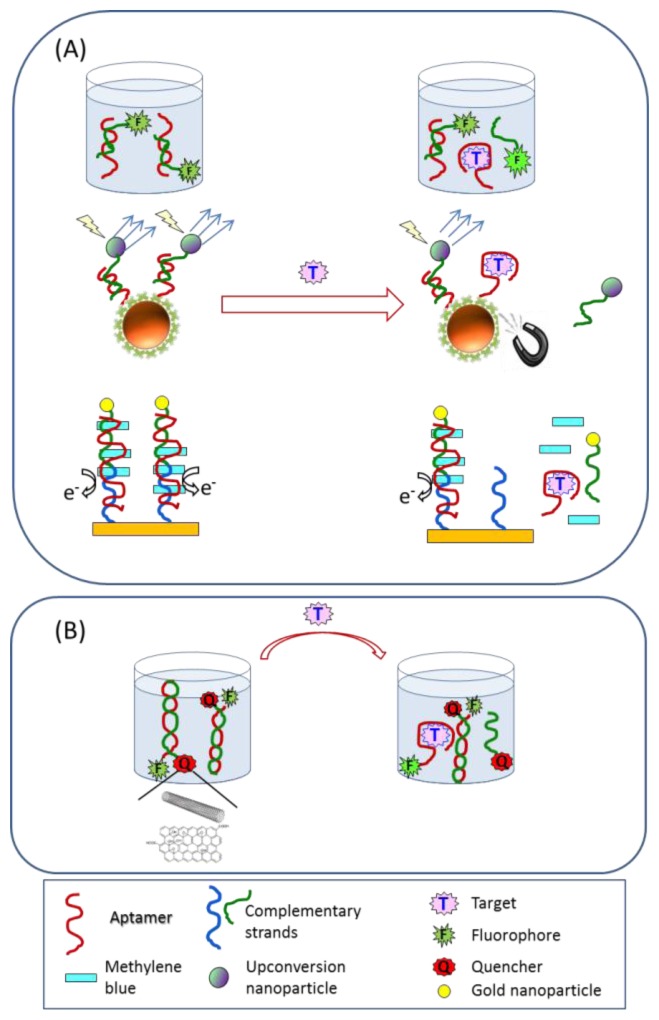
Displacement assay formats for micotoxin analysis in food (**A**) signal-off schemes; (**B**) signal-on schemes.

Mycotoxin detection is an active research area in the design of aptamer-based approaches. Among mycotoxins, ochratoxin A (OTA), a toxin produced by several fungus species of the genera *Penicillium* and *Aspergillus*, is one of the most dangerous contaminants in food commodities, especially cereal grains and related products, and it is certainly the biotoxin for which the greatest number of aptamer-based assays has been proposed. Beyond the classical competitive assay for small molecules that uses the immobilized aptamer as a receptor and gives rise to decreasing calibration curves [25], a great variety of target-induced strand displacement assays have been described. Initially, strategies for which the analytical signal decreases with the amount of target (signal-off assays) have been proposed (Figure 2A). Sub-nanomolar concentrations of OTA can be thus determined as a result of competition between the target and short oligonucleotides for binding to the aptamer, either in solution using short fluorescently labeled oligonucleotides and fluorescence polarization measurements [26], immobilizing the strand partially complementary to the aptamer on an electrode surface and using the voltammetric process of an intercalator as a signal [27] or combining magnetic separation of aptamer-functionalized magnetic nanoparticles and upconversion luminescent nanoparticles as labels of the release strand [28]. These assays, although rapid and selective, were not evaluated on food samples where they could suffer from matrix interferences and could be more prone to false positive results as a consequence of their declining signal.

**Figure 3.**
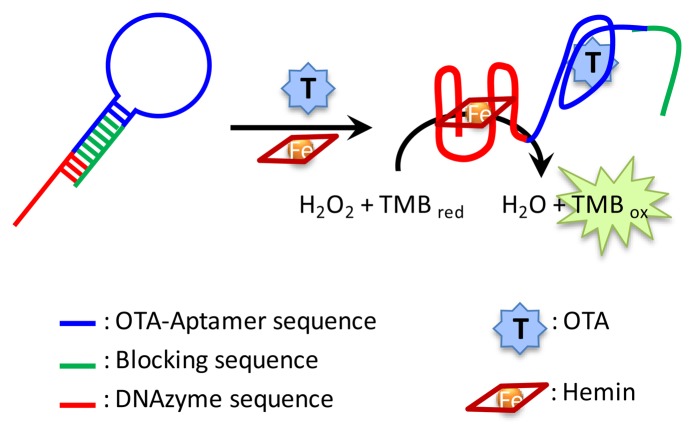
Hairpin-based colorimetric and homogeneous assay for the determination of OTA (adapted from [33]).

Different formats of signal-on assays based on fluorescence dequenching measurements have also been described (Figure 2B). In a typical assay the aptamer is labeled with a fluorophore which, in the absence of target, is quenched by a complementary DNA containing as quencher tetramethylrhodamine (TAMRA) [29], SWCNTs [30] or poly-vinyl pyrrolidone-protected GO [31]. Upon binding to its target the conformational change of the aptamer leads to the separation between the fluorophore and the quencher, generating an increase in the fluorescent signal related to the target concentration. The first of these tests was applied to the determination of OTA in spiked corn, with recoveries ranging from 83% to 106%. Additionally, the aptamer can be immobilized over nanoparticles such as gold nanoparticles, which act as the quencher of a fluorophore bound to a complementary DNA strand. The interaction with the target results in the displacement of this labeled-strand and in fluorescence recovery [32] showing high sensitivity for OTA detection with a limit of detection of 2 pg/mL. In the described assays, either the needs for aptamer immobilization or labeling may increase their complexity and cost. In an attempt to overcome these limitations a nucleic acid specially designed with a hairpin structure, which includes a peroxidase-mimicking DNAzyme, the sequence of the OTA specific aptamer and a blocking sequence, has been described [33]. In the absence of the target the blocking tail inactivates the DNAzyme, but the formation of the OTA-aptamer complex displaces this tail and the DNAzyme recovers its activity (Figure 3). The assay, although simple, shows limited sensitivity with a limit of detection of 0.1 ng/mL.

**Figure 4.**
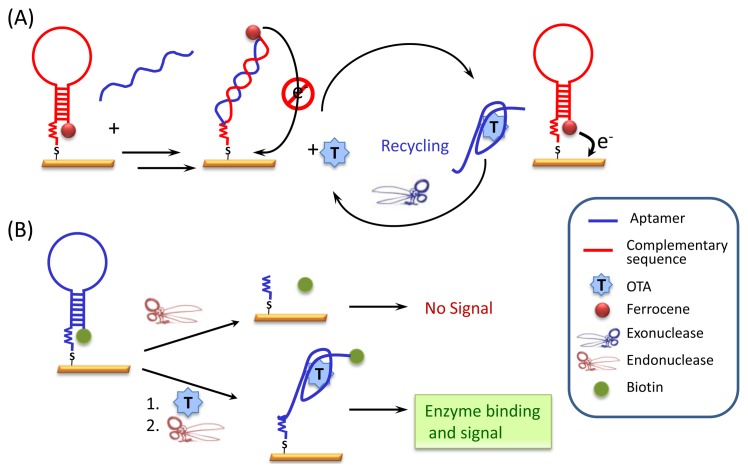
Ultrasensitive detection of OTA using (**A**) an exonuclease for target recycling; and (**B**) an endonuclease.

In order to achieve ultra-sensitive detection, various approaches for enhancing the transduction have been evaluated in combination with displacement assays. Some rely on the use of exo- or endonucleases to improve the analytical performance, following a clear trend since a few years. One possibility is target recycling by exonuclease cleavage of the aptamer (Figure 4A). For OTA detection in wheat starch, a DNA strand complementary to the aptamer, and designed to have a hairpin structure, is labeled with ferrocene, immobilized on the surface of a gold electrode, and hybridized with the aptamer. During the recognition event the aptamer is displaced from the surface and the complementary strand adopts the hairpin structure, forcing the electroactive label towards the electrode surface. An exonuclease selective for single strands finally digests the aptamer and recycles the target amplifying the signal [34]. Similarly, a restriction endonuclease selective to double-stranded DNA at a specific restriction site has been combined with a biotin-DNA aptamer immobilized on gold electrode (Figure 4B). In this case the nuclease cleaves the free aptamer, in its hairpin configuration with a double-stranded stem, and not the aptamer bound to the target, detecting as low as 0.4 pg/mL of OTA [35]. Finally, taking advantage of the nucleic acid nature of aptamers, isothermal amplification strategies (Rolling Circle Amplification) has been carefully designed to amplify the release sequence after target-aptamer interaction [36]. This approach has been tested for the determination of OTA in red wines achieving limits of detection of 0.2 pg/mL. Alternatively, the DNA fragment complementary to the aptamer can be used as a template for the polymerase chain reaction (PCR) resulting in a limit of detection of 1 fg/mL [37]. These strategies although extremely sensitive are often very time-consuming.

In the search for faster and simple detection methods useful for on-site detection, lateral flow strips are finding their place in the market for rapid, easy-to-read analysis. Commonly semiquantitative detection is provided without the need for readout equipment. However, the use of aptamers as the receptor of choice instead of antibodies is still at the beginning. An aptamer-based fluorescence strip that allows quantitative determination of ochratoxin A was recently reported. The determination is based on the competition between OTA and a DNA probe complementary to the aptamer immobilized on the detection zone. If there is OTA in the sample, QD-aptamer binds to OTA and cannot hybridize with the probe in the detection zone. This signal-off sensor permits fast quantification of the mycotoxin contamination in wine. For rapid screening, only a UV-pen-light is needed [38].

The use of novel materials and multiplexing detection is used for designing a fluorescence resonance energy transfer based assay. This work developed a platform for simultaneous detection of aflatoxin M1 and ochratoxin based on aptamer-modified lanthanide-doped upconversion nanoparticles and GO. The aptamer-NP bound to the mycotoxins cannot bind to GO and its fluorescence intensity cannot be quenched. This multiple sensor has been tested to detect mycotoxins in maize [39].

Electrochemical label-free formats have been also reported. Polyaniline was electrogenerated to act as both immobilization layer for aptamer and the signaling compound. The blocking of the redox activity of the polymer after binding of increasing concentrations of aflatoxin M1 resulted in a voltammetric signal-off device. The sensor can be regenerated by displacing aflatoxin from their complex on the surface by a high concentration of aptamer in solution for several hours, which is not very practical [40]. Polyaniline also constituted the anchoring bridge molecule to covalently attach OTA to ITO electrodes by preforming a mixed Langmuir-Blodgett with stearic acid. The addition of the mycotoxin caused a decrease in the impedance of the interface, which was attributed to the G-quartet structure adopted by the anti-OTA aptamer that may facilitate the electron-transfer through well-ordered chains of conducting polyaniline [41].

Bioterrorist threats such as abrin or ricin toxins are attractive targets for aptamer selection because of their extreme toxicity. In contrast to mycotoxin, they are large molecules so they could benefit from the sandwich strategy if two binding sites for aptamer are present or two distinct aptamers are available. A direct uncomplicated luminescent assay for abrin toxin using a ruthenium complex was developed although no food matrix but human serum was tested [42]. Surface Enhancement Raman Spectroscopy (SERS) using silver dendrites modified with ricin specific aptamer permits detection of ricin in several liquid food matrices such as juices, lemonade and milk [43].

## 4. Allergens

Food allergy is an adverse, abnormal immune-mediated reaction to a certain food or food ingredient that occurs in susceptible individuals that often requires a strict avoidance of the offended component. Food sensitivity also includes food intolerance, which is clinically undistinguishable from allergy but is a non-immune mediated reaction [44]. The incidence of these disorders is difficult to assess and a discrepancy between people self-perceived as food intolerant (up to 25%) and the food confirmed cases (less than 3%) is apparent. In general a prevalence of 1%–3% in the USA [44] and EU [45] has been reported. The increasing intake of packing foods has compelled to governments to subject the food industry to more and more stringent scrutiny in order to control the allergen content. Given that the real problem with allergen is the presence of allergenic residues in food that naturally do not contain them due to cross-contamination with other production chains, food manufacturers have invested a huge amount of resources to diminish and control the allergen content. Additionally, since 2003 EU regulations oblige to compulsory labeling foodstuff containing a list of allergens [46] that successive amendments have raised to 14 groups of food (the worldwide recognized big eight: milk, eggs, peanuts, tree nuts, soy, wheat, fish, and shellfish, plus celery, mustard, sesame, sulphur dioxide and sulphites, lupin and molluscs) [47,48]. Differences not only in the allergen list but also in the content threshold for labeling across the globe make difficult the development of analytical methods universally applicable [49].

Most allergens are proteins that can suffer significant modification during processing, sometimes reducing their allergenicity. In any case, the variability of allergen presentation and their interaction with food matrix is a challenge for the development of methods of analysis that should detect the injurious target in all circumstances.

Since the increase in allergen awareness and regulations, a great variety of commercial analytical tools have been developed [49]. Most of them rely on immunoassays, both competitive for hydrolyzed food and sandwich formats for complete proteins. Recently, the selection of aptamers for this group of ingredients is emerging. There are just a few aptamers selected to bind allergenic proteins, and only a couple of them have been tested for analytical purposes. One of them targeted one of the allergenic proteins from lupin, a recently incorporated allergenic source in European legislation, which lacks highly specific recognition elements. The protein β-conglutin was the target protein for the development of a competitive ELISA-type assay showing that the protein can be determined at concentrations higher than 153 nM in aqueous media, but the method was not challenged to demanding food matrices [50].

The aptamer developed to bind the protein Ara H1 from peanut has been used as a capture reagent in a sandwich assay using fiber optic coated surface plasmon resonance (SPR). An antibody acted as secondary recognition element but the wavelength shift was too small and an amplification scheme was implemented by using protein A-modified gold nanoparticles. Candy bar matrix was fortified with the allergen and diluted five times to decrease the viscosity of the sample that diminishes the sensitivity. The mixed sandwich approach was less sensitive than a previous one using two antibodies. As authors recognize the ultimate goal is the detection of allergen from real samples where extraction problems have to be carefully considered [51].

Recently aptamers against an immunotoxic peptide from gliadin from wheat that also recognize celiac disease related proteins from barley, rye and oat were selected [52]. A competitive electrochemical magnetoassay was developed without cross-reactivity with non-triggering celiac disease proteins from soy, rice or maize [53].

## 5. Other Growing Food Concerns

Human development and activities generates a great variety of novel compounds without regarding the consequences on environmental or human health at long term. In spite of the efforts, many of them can easily enter to the water or food chain. Society is more and more aware of the risks associated with an, in principle, pleasant and inevitable task such as eating, and urges authorities and industry to establish control and surveillance measures to guarantee the quality of the food even for compounds whose potential hazard is under controversy. In this context, emerging pollutants are generally considered those compounds that are not included in regulations currently in force [54] or even are under study as potential hazards for the environment and human health. In this section we join together contaminants of anthropogenic origin either unregulated or already regulated but involved in special concerns such as veterinary residues.

Among this wide range of substances, contaminants of industrial origin or pharmaceuticals can be found. Among the former, pesticides, dyes, adulterants or packaging materials that can migrate to food are more often found in food. Among pharmacological residues those of veterinary uses can be found in meat and milk because they are massively used to prevent animal infection and can pass through the food chain to human being when the waiting time is not long enough before commercialization. Additional pharmaceutical from human therapies can also reach environmental waters from home sewage. This kind of water contaminants are not removed in wastewater treatment plants, so they finally enter the drinking water systems.

Despite there are many aptamers against pharmaceuticals, most assays or sensors have been tested in clinical samples. Only a few of them have been applied in food. The first aptasensor reported for antibiotic detection also pioneers the use of modified-RNA aptamers to overcome the natural degradation of this reagent in biological fluids for analytical purposes. Since neomycin B is a small molecule, a displacement scheme was developed by covalently attaching the antibiotic to a SAM on Au electrodes and subsequent immobilization of the aptamer by affinity to build the sensing phase. The presence of increasing concentrations of the antibiotic displaces the aptamer from the surface decreasing the electron transfer resistance measured by faradaic impedance spectroscopy without noticeable cross-reactivity with other structurally related compounds. This label-free aptasensor was successfully applied to spiked milk samples that require a pretreatment because of the association of neomycin to milk serum proteins [55]. Labeling strategies have been also reported. A strand release electrochemical method was employed to determine chloramphenicol, a broad-spectrum antibiotic banned in many countries for use in food-producing animals than can enter the food chain not only through its unauthorized uses but also as a consequence of its employment in human treatments. An anti-chloramphenicol aptamer was immobilized on the surface of a gold electrode and hybridized with its complementary biotinylated strand. When the recognition event takes place, the complementary strand is released and the remaining biotinylated strands are labeled with a streptavidin-enzyme conjugate. Consequently, the analytical signal decreases when the concentration of the drug increases. This biosensor was tested in spiked honey after an extraction step. Recoveries from 84.4% to 102% were found and the results compared well with a more costly LC-MS/MS method [56]. Tetracyclines are another group of antibiotics whose abuse in veterinary practice to treat infectious diseases is causing accumulation in meat, milk, honey, *etc.*, and therefore pose a health threat. Chromatography is the most common technique for their analysis as a group, in spite of requiring extensive sample pretreatment. The individual detection of each member is also possible using specific recognition reagents such as aptamers with the promise of a more rapid detection. However, methods developed so far, a voltammetric aptasensor [57] and a conventional competitive assay on microtiter plates [58], still require a rather complex pretreatment of the milk samples. A limited degree of cross-reactivity with other tetracycline members was found in both cases. Microfluidic platforms using aptamers as receptors were also proposed for the analysis of two antibiotics: ampicillin and kanamycin A. Non-faradaic impedance at a single frequency allowed the continuous monitoring of the binding. Due to the unspecific nature of the impedance measurements multiplexing is not possible. The ampicillin enrichment of non-fat milk was carried out after dilution of the sample and directly applied to the sensor. This is not a realistic approach to take into account the real interaction between the drug and the matrix [59].

Hormones are chemicals naturally produced by animals and humans, but there are also synthetic and natural hormones used as pharmaceuticals to increase the profitability of meat and dairy industries, especially steroid hormones. This kind of hormones such 17β-estradiol are endocrine disruptors that may affect the reproduction of humans and animals and induce tumors. Their levels in wastewaters have to be controlled. An evanescent wave fiber-optic aptasensor that requires a portable compact instrumentation was proposed for the detection of this compound in a competitive format. The analyte, estradiol, was incubated with a fixed amount of fluorescently-labeled aptamer and pumped towards the sensing cell where estradiol is covalently attached. The analytical signal was monitored in continuous and the sensor is easily regenerated. It was applied to spiked wastewater treatment effluents [60].

Since a decade, monitoring of contaminants of industrial origin has shifted from environmental samples to marine food and dairy products [61], which indicates the increasing alarm that the presence of these compounds in food is causing. As an example, the presence of the dye malachite green, an antifungal, antiparasitic, and antiprotozoan agent used in aquaculture since 1936, has been detected using a fluorimetric homogeneous aptamer-based assay in fish samples. The increase in fluorescence of the target when it is bound to the aptamer was measured. To test the assay in salmon tissues, the highly persistent leucoform of the dye was spiked. A solid-phase extraction and an oxidation protocol compatible with the assay were needed [62].

Another important group of priority and emerging pollutants in food is pesticides. The implementation of new and more restrictive regulations for these substances, which are widely used to control pests and protect crops, has driven the development of improved analytical methods, mainly based on gas and liquid chromatography with different detectors. These methods are able to detect a large number of compounds at extremely low levels, but they are expensive and time-consuming. To reduce the number of samples to be processed, screening methods have been employed, among them enzyme- inhibition based biosensors and immunosensors. One could think of aptamer-based assays as an alternative to these screening methods. In fact, some aptamers have been developed against chemical pesticides, but as far as we know only one example of aptasensor for the detection of the insecticide acetamiprid has been reported. In this case, a label free strategy, based on conventional mixed SAM and impedance detection, has been proposed. Nanostructuring with gold nanoparticles increased the effective electrode area. The aptasensor was tested in samples of wastewater and tomatoes [63]. However, there are so many pesticides in use, with so different structures, that the development of specific aptasensors does not seem a good alternative to solve the problem of pesticides screening in foods.

Finally, materials used in food packaging are another source of chemical residues than can pose a health threat in some situations. This is the case of melamine, commonly used in plastics that may be in contact with food so its maximum migration level is regulated. In addition, its use as adulterant was noticed in dairy product for infants from China because of its high content in nitrogen to replace proteins. This triggered a food alarm and compelled European agencies to implement firstly a ban on dairy Chinese imports and then a regulation on the maximum recommended level in these products. Catalytic optical label-free methods were developed to detect melamine. Nanogold and nanosilver particles can adsorb aptamers which precludes their aggregation. In the presence of melamine, aptamers are released from nanoparticles, yielding metal aggregates that cannot catalyze the Fehling reaction. The decrease in the resonance scattering intensity at 614 nm from the Cu-based catalytic system is the analytical signal for both approaches that were tested in milk samples [64,65]. The aptamer for melamine was selective against some ions and proteins but interestingly, it is a polyT sequence, which is a very unusual result from SELEX. Using scientific database we were unable of finding the SELEX for the selection of this aptamer. Another compound that can migrate from packaging and is a suspected endocrine disrupting agent was also the target of SWCNT-field-effect transistor (FET) using aptamers as recognition element on Au surfaces. The binding of bisphenol A, a neutral compound, to its aptamer did not decrease the conductance of FET, so a sandwich strategy using a detection aptamer was implemented. To further improve the sensitivity from 1 pM to 10 fM, the aptamer was labeled with biotin, and coupled to enzymatic amplification. No real sample analysis was reported [66].

As mentioned above, pharmaceuticals and other anthropogenic contaminants accumulate in water because there are not specific treatments in wastewater plants for their removal. Aptamer can also be seen as efficient and selective capture elements and their use for preconcentration or clean-up systems can be advantageously exploited. With this regard, the activity of aptamers in continental water may be impaired because of the need of specific buffer composition for adequate performance. Therefore, a protecting vehicle could provide the proper environment to maximize their activity when introduced in the sample. Liposomes filled with aptamers and their binding buffers were designed for removal of three small organic contaminants, 17β-estradiol, bisphenol A and oxytetracycline, in drinking water. A capture efficiency 80% superior to that of aptamers in solution was achieved, although elimination of a combination of contaminants with a mixture of liposomes carrying each aptamer was less efficient [67].

The easiness of separation and high binding capability of magnetic beads (MB) have converted this material into an ideal support for this kind of applications, especially for commercial kits. In the particular case of pathogens, combination of aptamagnetic enrichment with the exquisite sensitivity of PCR allows extreme sensitivities even in complex food matrices. Several examples of successful aptamagnetic separation were reported for *Salmonella* [68,69] and *Listeria* [70]. In all cases, quantification was achieved by means of real time PCR amplification of a specific gen of corresponding pathogen after DNA extraction from captured cells. In the case of *Listeria*, the analytical features were similar to those achieved with an immunomagnetic separation, except for a somehow better capture efficiency. However, the capture efficiency of both affinity elements was poor when testing large volume samples even using a recirculating unit. This was attributed to the deleterious effect of flow that can be overcome if aptamers with higher affinity are used [69]. Binding in solution and following capture of the complex between *Salmonella typhimurium* and a biotinylated aptamer on streptavidin-MBs was also reported [68] but it was not applied to real samples. Another anti-*Salmonella typhimurium* aptamer-modified-MB system, however, was directly tested in fecal and artificially contaminated carcass chicken rinse samples. A low level of contamination 10–100 cfu in 9 mL was obtained in these difficult samples. Since PCR methods are not applicable to non-pathogenic targets, other techniques such as confocal microscopy [71] or capillary electrophoresis [72] have to be selected for detection. In this way, good recoveries were reported for the isolation of zearalenone from spiked beer at high levels, above 10^−5^ M, and dilution with buffer for measuring [71]. Magnetite nanospheres covered by a poly(styrene/acrylamide) copolymer were synthesized for mycotoxin capture. The amine groups on its surface were turned into carboxylic acid for aptamer anchoring through hydrazine activation. Recoveries of OTA from 67% to 90% were found in spiked wheat flour, coffee and cereal products. HPLC was coupled to this capture method for quantification [73]. Similarly, aptamer-modified glass microbeads selectively isolated 17β-estradiol in the presence of other hormones and antibiotics and this enrichment was coupled to the chromatographic determination of the hormone in spiked river water [74].

Recently the preparation of aptamer-based oligosorbent cartridges for solid-phase extraction of OTA prior to fluorescent [75] or HPLC with fluorescent detection has been proposed [76,77]. OTA, naturally present in wheat samples with certified OTA concentrations and other cereals, was concentrated and separated using a specific aptamer covalently attached to a resin. This oligosorbent was compatible with up to 20% of methanol and lower dilution of target in binding buffer than antibody-based separation approaches [75]. An overall mean recovery of 79% from wheat samples spiked at 0.5–5 ng/g level [76] and 89% from beer spiked at 1–3 ng/g level [77] was found with this kind of cartridges. These aptamer oligosorbents compared reasonably well with their analogues based on antibodies. Quite similar recoveries were obtained although in the case of wheat analysis the sensitivity of the overall analytical method was slightly superior. The main advantage is the simple and rapid reusability achieved with aptamers. In contrast to antibodies that require days to recover their natural conformation, aptamers can recover their activity within minutes.

## 6. Concluding Remarks

In this feature, an overview of the considerable variety of aptamer-based assay systems that can be applied to the analysis of food, and how food chemists, regulatory agencies, and quality control laboratories aimed to ensure food safety might take advantages of these advances, has been presented.

Aptamer-based assays, in particular aptasensors in combination with nanomaterials, surface fabrication techniques and integrated micro-fluidic devices able to offer platforms with a high degree of automation and multiplexing capabilities, may become a viable option for the development of more powerful, cleaner and cheaper analytical methods for the detection of pathogens, natural toxins, allergens and other emerging groups of contaminants in food. Among the different detection techniques, electrochemical transducers are the closest to fulfill all the requirements of portability, accuracy, ruggedness and ease of use that commercial devices must meet, especially when coupled to label-free strategies such as the case of potentiometric and impedimetric devices.

However, only preliminary assays based on a reduced number of suited aptamers and at a purely academic stage have been described. There is a clear need for selection of new aptamers against allergens, pathogens and the great variety of toxic residues that can be found in food, which is continuously increasing. Selecting new aptamers should occur in parallel with the development of analytical assays.

Another important challenge that still remains in aptamer-based food analysis is the simplification of sample preparation, which limits the speed of attainment of results. Most of the described assays have proved the quantification of targets in aqueous solutions and only a few face the analysis of spiked samples. The development of online sample preparation systems will also be required in order to facilitate the development of commercial devices.

Nonetheless, this is a very active area of research and new and exciting developments will be achieved in the near future where we will see the start of the race to bring to market aptamer-based assays for different targets of importance in food safety control. This will require overcoming the resistance of the agri-food sector, a conservative sector mainly relying on well-known processes and not focused on technology. But as food analysts are facing increasingly complex challenges, they will need the best available technology and this is where aptamers will find their niche.
